# Polarization of membrane associated proteins in the choroid plexus epithelium from normal and *slc4a10* knockout mice

**DOI:** 10.3389/fphys.2013.00344

**Published:** 2013-11-27

**Authors:** Inga B. Christensen, Tua Gyldenholm, Helle H. Damkier, Jeppe Praetorius

**Affiliations:** Department of Biomedicine, Faculty of Health, Aarhus UniversityAarhus, Denmark

**Keywords:** sodium hydrogen exchanger, sodium bicarbonate cotransporter, epithelial polarization, cytoskeleton, choroid plexus, cerebrospinal fluid

## Abstract

The choroid plexus epithelium (CPE) has served as a model-epithelium for cell polarization and transport studies and plays a crucial role for cerebrospinal fluid (CSF) production. The normal luminal membrane expression of Na^+^,K^+^-ATPase, aquaporin-1 and Na^+^/H^+^ exchanger 1 in the choroid plexus is severely affected by deletion of the *slc4a10* gene that encodes the bicarbonate transporting protein Ncbe/NBCn2. The causes for these deviations from normal epithelial polarization and redistribution following specific gene knockout are unknown, but may be significant for basic epithelial cell biology. Therefore, a more comprehensive analysis of cell polarization in the choroid plexus is warranted. We find that the cytoskeleton in the choroid plexus contains αI-, αII-, βI-, and βII-spectrin isoforms along with the anchoring protein ankyrin-3, most of which are mainly localized in the luminal membrane domain. Furthermore, we find α-adducin localized near the plasma membranes globally, but with only faint expression in the luminal membrane domain. In *slc4a10* knockout mice, the abundance of β1 Na^+^,K^+^-ATPase subunits in the luminal membrane is markedly reduced. Anion exchanger 2 abundance is increased in *slc4a10* knockout and its anchor protein, α-adducin is almost exclusively found near the basolateral domain. The αI- and βI-spectrin abundances are also decreased in the *slc4a10* knockout, where the basolateral domain expression of αI-spectrin is exchanged for a strictly luminal domain localization. E-cadherin expression is unchanged in the *slc4a10* knockout, while small decreases in abundance are observed for its probable adaptor proteins, the catenins. Interestingly, the abundance of the tight junction protein claudin-2 is significantly reduced in the *slc4a10* knockouts, which may critically affect paracellular transport in this epithelium. The observations allow the generation of new hypotheses on basic cell biological paradigms that can be tested experimentally in future studies.

## Introduction

The cellular monolayer of the choroid plexus produces the majority of the cerebrospinal fluid (CSF) by highly efficient transepithelial movement of solutes and water molecules (Damkier et al., 2013). CSF secretion relies on the concerted action of a variety of channels, pumps, and cotransporters situated in either the luminal plasma membrane, the basolateral plasma membrane, or in the tight junctions of the choroid plexus epithelium (CPE). The surface area of the luminal membrane of CPE is enlarged by extensive microvilli and the basolateral membrane is characteristically enlarged by basolateral infoldings referred to as the basal labyrinth. The distribution of most of the proteins in the CPE is highly conserved among studied mammalians, and even among some non-mammalian vertebrates, and is strikingly different from most other transporting epithelia. Most prominently, the Na^+^,K^+^-ATPase is expressed exclusively in the luminal membrane in the CPE (Zeuthen and Wright, 1978; Masuzawa et al., 1984). This is also the unusual localization of NKCC1 (Plotkin et al., 1997; Wu et al., 1998), whereas other proteins such as AE2 have a normal membrane distribution (Lindsey et al., 1990). A variety of Na^+^-dependent acid/base transporters are expressed in the CPE and are expected to play central roles in CSF secretion and its pH regulation (Damkier et al., 2010).

The Na^+^ driven acid/base transport proteins belong to two gene families, namely the solute carrier families 4 and 9. The Na^+^-dependent HCO^−^_3_ transporters are encoded by *slc4a4* (NBCe1), *slc4a5* (NBCe2), *slc4a7* (NBCn1), *slc4a8* (NDCBE), and *slc4a10* (Ncbe/NBCn2). Of these, NBCe2 is expressed in the luminal membrane of the CPE (Bouzinova et al., 2005), Ncbe in the basolateral membrane (Praetorius et al., 2004), and NBCn1 in either the luminal or basolateral membrane depending on the species or strain (Praetorius et al., 2004; Praetorius and Nielsen, 2006). A recent report suggests that the *slc4a11* gene product (NaBC1) is a Na^+^ permeable pH_*i*_ regulator (Ogando et al., 2013). NaBC1 is expressed in the luminal membrane of the CPE (Damkier et al., 2007). The Na^+^/H^+^ exchangers are encoded by the *slc9a1-9* genes. Only NHE1 (*slc9a1*) is expressed in the CPE, where it is confined to the luminal membrane (Damkier et al., 2009). This is a highly atypical position for this almost ubiquitously expressed protein.

Membrane proteins are sorted and trafficked to the various membrane domains from the trans-Golgi apparatus and through common recycling endosomes. The membrane proteins are delivered to the specific plasma membrane domains by vesicular transport, and retained in the membrane by anchoring proteins linking the membrane protein to the cytoskeleton (Bryant and Mostov, 2008; Mellman and Nelson, 2008). In most epithelia, the Na^+^,K^+^-ATPase is linked to the spectrin cytoskeleton through ankyrins and thus, all these proteins accumulate in the basolateral cell domain (Morrow et al., 1989; Nelson and Hammerton, 1989). In the CPE, the general spectrin cytoskeleton and undefined ankyrins are found primarily near the luminal membrane as opposed to most other polarized epithelia (Marrs et al., 1993; Alper et al., 1994). It is unknown whether the atypical distribution of the membrane proteins in the CPE is caused by cell type specific distribution of cytoskeletal proteins, anchoring proteins, membrane proteins, or other factors. Candidate proteins include the various spectrin isoforms, ankyrins, catenins, adducins, ERM proteins, as well as the Na^+^,K^+^-ATPase, NKCC1, and NHE1.

We have previously shown that the distribution of certain membrane proteins in the CPE is prominently altered in *slc4a10* knockout mice (*slc4a10* ko) compared to wild type (wt) littermates: In *slc4a10* ko mice, NHE1 is localized to the basolateral membrane, and ezrin that usually anchors NHE1 to the actin cytoskeleton, is distributed within the cytoplasm and less in the luminal membrane (Damkier et al., 2009). In the same mouse model, the expression levels of Na^+^,K^+^-ATPase and the water channel AQP1 is markedly decreased, while NBCn1 and NBCe2 expression is unaffected (Damkier et al., 2009; Damkier and Praetorius, 2012).

The many unknown aspects regarding the anchor protein distribution and possible interactions between membrane proteins and anchor proteins warrant a more systematic and exhaustive approach to uncover the causes and consequences for the CPE polarization. In such studies, we regard the *slc4a10* ko mouse model a useful tool. In the current study, we aimed to (1) define the spectrin and ankyrin isoforms in the CPE, (2) determine the distribution of E-cadherin, adducin, and catenin proteins, and (3) describe the cellular polarization of major membrane proteins and their anchoring proteins in CPE from *slc4a10* ko and wt mice.

## Materials and methods

### Animals

The breeding and genotyping of mouse models deficient in *slc4a10* have previously been described (Jacobs et al., 2008). Mice were bred on c57bl/6 background, and female and male mice littermates aging 4–5 weeks were used in an ~50:50 ratio. All procedures conformed to Danish animal welfare regulations. The authors are licensed to breed the mouse strains by The Animal Experiments Inspectorate, Ministry of Food, Agriculture and Fisheries (j.n. 2012-15-2935-00004).

### Immunohistochemistry

All mice were perfusion fixed in succession via the heart with 3% paraformaldehyde in a phosphate-buffered salt solution (PBS, in mM: 167 Na^+^, 2.8 H_2_PO^−^_4_, 7.2 HPO^2−^_4_; pH 7.4). After fixation the brain was removed, post-fixed for 2 h, dehydrated, and embedded in paraffin wax, enabling 2 μm sections to be cut using a rotary microtome (Leica). The sections were de-waxed and stepwise rehydrated, before epitopes were retrieved by boiling the sections in 10 mM Tris buffer (pH 9) with 0.5 mM EGTA. The epitopes were quenched with 50 mM NH_4_Cl in PBS, and unspecific binding was blocked by washing with 1% BSA in PBS with 0.2% gelatin and 0.05% saponin. Sections were incubated overnight at 4°C with primary antibody diluted in 0.1% BSA in PBS added 0.3% Triton X-100. Primary antibodies are listed in Table 1, and positive control tissues included kidneys, brain, vasculature, and red blood cells (not shown).

**Table 1:** **Primary antibodies applied in the study**.

**Target**	**Antibody**	**Host**	**Source**
γ-actin	LS-C34852	Sheep	LifeSpan
α-adducin	sc-25731 (H-100)	Rabbit	Santa Cruz Biotech
β-adducin	sc-25732 (H-120)	Rabbit	Santa Cruz Biotech
γ-adducin	sc-25733 (H-60)	Rabbit	Santa Cruz Biotech
Anion exchanger 2	c-terminal AE2	Rabbit	Stuart-Tilley (Stuart-Tilley et al., 1994)
Ankyrin-1	sc-12733 (8C3)	Mouse	Santa Cruz Biotech
Ankyrin-2	LS-C11198	Mouse	LifeSpan
Ankyrin-3	sc-28561 (H-215)	Rabbit	Santa Cruz Biotech
α-catenin	LS-B4457	Goat	LifeSpan
β-catenin	sc-7199 (H-102)	Rabbit	Santa Cruz Biotech
Claudin-2	SAB4503544	Rabbit	Sigma
E-cadherin	610181	Mouse	BD Biosciences
Ezrin	sc-6409 (C-15)	Goat	Santa Cruz Biotech
Moesin	ab50007	Mouse	Abcam
α1 Na^+^,K^+^-ATPase		Mouse	Kashgarian (Kashgarian et al., 1985)
β 1 Na^+^,K^+^-ATPase		Rabbit	Martín-Vasallo (Gonzalez-Martinez et al., 1994)
αI-spectrin	LS-C137722	Rabbit	LifeSpan
αII-spectrin	sc-46696 (C-11)	Mouse	Santa Cruz Biotech
βI-spectrin	LS-C138700	Rabbit	LifeSpan
βII-spectrin	sc-28272 (H-125)	Rabbit	Novus
βIII-spectrin	sc-28273 (H-70)	Rabbit	Santa Cruz Biotech
βIV-spectrin	LS-B5099	Goat	LifeSpan
βV-spectrin	sc-104664 (C-13)	Goat	Santa Cruz Biotech
Syntaxin-3	sc-47437 (N-17)	Goat	Santa Cruz Biotech

For bright-field light microscopy, the sections were incubated in horseradish peroxidase-conjugated secondary antibodies (DAKO, Glostrup, Denmark) diluted in PBS with BSA and Triton X-100. The staining was visualized using 0.05% 3,3′-diaminobenzidine tetrahydrochloride dissolved in PBS with 0.1% H_2_O_2_. Mayer's hematoxylin was used for counterstaining, and the sections were dehydrated in graded alcohol and xylene. Finally, sections were mounted in Eukitt mounting medium (O. Kindler, Freiburg, Germany). The images were acquired using a Leica DMRE bright-field microscope equipped with a Leica DM300 digital camera. For fluorescence visualization of the primary antibodies, AlexaFlour 488- or 555-coupled donkey anti-goat, -sheep, -rabbit, or -mouse secondary antibodies (Invitrogen) were used, and cell nuclei were visualized using Topro3 counterstaining (Invitrogen). Sections were mounted with a coverslip in Glycergel antifade medium (DAKO) and analyzed using a Leica DMIRE2 inverted microscope with a TC5 SPZ confocal unit using ×63/1.32 NA or ×100/1.4 NA HCX PI Apo objectives with 8-bit depth for illustration of localization and colocalization, while 12-bit image depth was applied for fluorescence semiquantitation.

### Semiquantitation of immunofluorescence sections

Specific protein abundance was investigated by quantifying the immunofluorescence intensities from confocal micrographs. All tissues were carefully handled in parallel from the time of fixation throughout embedding, sectioning, staining, and imaging. To avoid saturation of the photomultiplier, the intensity dynamic range (gain and offset) was adjusted to span the intensities of the most intense sample for each antibody. Images were acquired in the focal plane with the highest signal intensity using fixed settings for magnification, laser power, gain, image depth, offset, and averaging for all images with a given antibody.

The immunofluorescence intensities of the stained choroid plexuses were quantified from 12-bit gray scale images using Image Pro (Media Cybernetics). For each image, the area of interest was manually defined to avoid counts from non-choroidal tissue or artifacts. For all quantifications, the number of cell nuclei within the area of interest was automatically counted with fixed settings for minimal cell nucleus area, density/intensity signal, smoothing, and intensity range. The sum of immunofluorescence intensities was then divided by the number of nuclei to normalize for differences in choroid plexus tissue size among the sections. All analyzed images were from 4th ventricle choroid plexus, and data from the right and left brain sections were averaged for each animal when possible. In bar graphs, data are normalized to the mean wild type fluorescence signal. Where indicated, linescan intensity profiles were generated from fluorescence micrographs using Image Pro. The lines of interest are marked in white on the respective micrographs and represent only examples of juxta-labyrinth profiles from the indicated line.

### Statistical analysis

Data is expressed as means ± sem. A non-parametric test (Mann–Whitley–Wilcox rank sum test) was used to compare two groups. Values of *p* < 0.05 were considered statistically significant. Each *n* represents one animal.

## Results

### The choroid plexus epithelium expresses ankyrin-3 and αI-, αII-, βI-, and βII-spectrins

**Figure 1:**
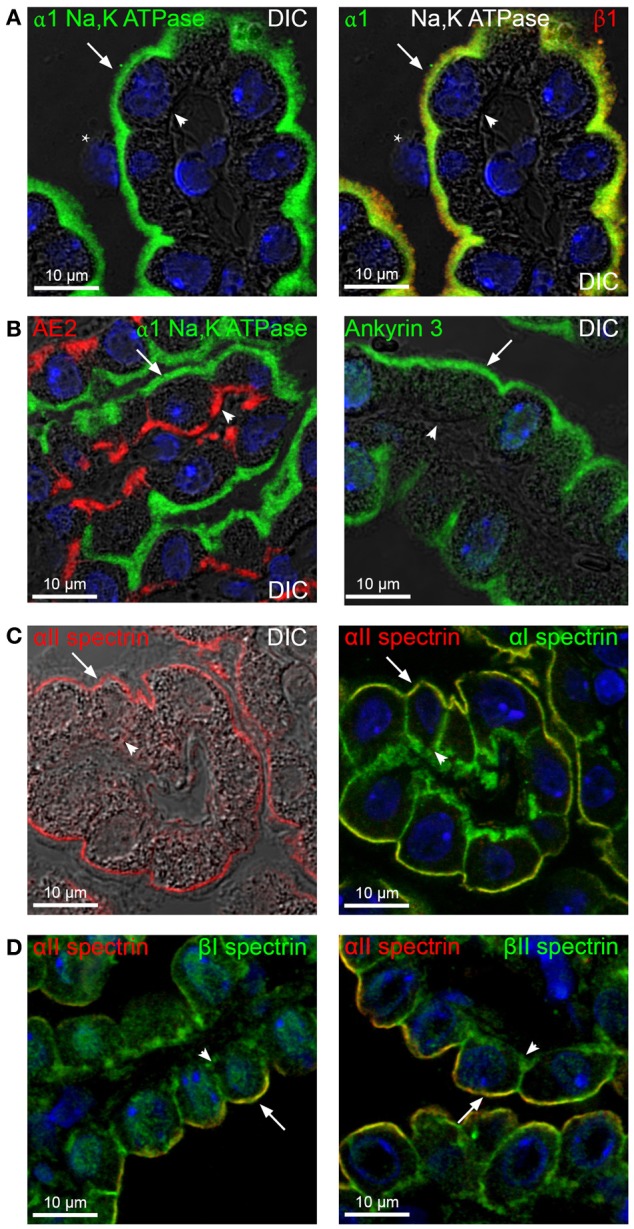
**Expression of spectrins and ankyrins in the choroid plexus epithelium**. Mouse brain sections were immunostained for Na^+^,K^+^-ATPase α1 subunit, and ankyrins 1-3, αI-II and βI-V spectrins (in green), as well as Na^+^,K^+^-ATPase β1 subunit and AE2 (in red). Yellow color indicates co-localization. **(A)** Immunohistochemical staining for Na^+^,K^+^-ATPase α1 subunit overlaid on differential interference contrast image (DIC, left panel). The same section was doublestained for Na^+^,K^+^-ATPase α1 and β1 subunits (right panel). ^*^ marks an apparent macrophage (Kolmer cell). **(B)** Double immunofluorescence staining for AE2 and Na^+^,K^+^-ATPase α1 (left panel), and immunohistochemical detection of ankyrin-3 with DIC (right panel). **(C)** Immunostaining for αII-spectrin overlaid on the corresponding DIC image (left panel). Double immunofluorescence detection of αII-spectrin and αI-spectrin (right panel). **(D)** Immunolabeling for βI- and βII-spectrins (left and right panel, respectively) in doublelabeling with αII-spectrin. Arrows indicate the luminal membrane, while arrowheads indicate the basolateral membrane. Cell nuclei were visualized by Topro nuclear staining (blue).

The Na^+^ independent HCO^−^_3_ exchanger AE2, as well as the Na^+^,K^+^-ATPase, is typically linked to the spectrin cytoskeleton via anchoring proteins, such as ankyrins, in the basolateral membrane. In the CPE, however, the α1 subunit of the Na^+^,K^+^-ATPase is expressed in the luminal brush border, along with the β1 subunit of the Na^+^,K^+^-ATPase (Figure 1A, left and right panels, respectively). AE2 is located in the basolateral plasma membrane, similar to other epithelia (Figure 1B, left panel).

As the expression of spectrin and ankyrin isoforms in the CPE have not been determined, a panel of antibodies reacting specifically against ankyrins 1-3, spectrins αI-II, and spectrins βI-V were employed for immunohistochemical analysis of the CPE. As illustrated in Figure 1B (right panel), ankyrin-3 immunoreactivity was predominantly observed in close proximity to the luminal membrane and in microvilli. No other ankyrins were localized to the CPE with the applied antibodies but did label control tissues (not shown).

The αII-spectrin immunoreactivity was mainly observed in the luminal membrane domain (Figure 1C, left panel). However, αI-spectrin immunoreactivity was most prominent in relation to the basal labyrinth and to a lesser extent in the luminal membrane domain of the CPE cells (Figure 1C, right panel). βI- and βII-spectrin specific antibodies also produced a mainly luminal staining pattern with minor staining in relation to the lateral membrane and basal labyrinth (Figure 1D, left and right panels, respectively). The βII-spectrin labeling was also observed in a supra-nuclear location, where the microtubule organizing center is situated in CPE cells (see Figure 8). No other spectrin antibodies reacted with the CPE (not shown). The CPE expresses ankyrin-3, αII-spectrin, βI-spectrin, and βII-spectrin in high abundance beneath the luminal membrane and the three spectrins at a lesser extent in proximity to the basal labyrinth. In contrast, the fourth spectrin expressed in the CPE, αI-spectrin, shows an opposite expression pattern, with most expression in the basolateral labyrinth and minor in the luminal membrane. In regard to the different cellular localizations, AE2 is unlikely to bind ankyrins in the choroid plexus.

### E-cadherin, α- and β-catenin are co-expressed in the basolateral domain

**Figure 2:**
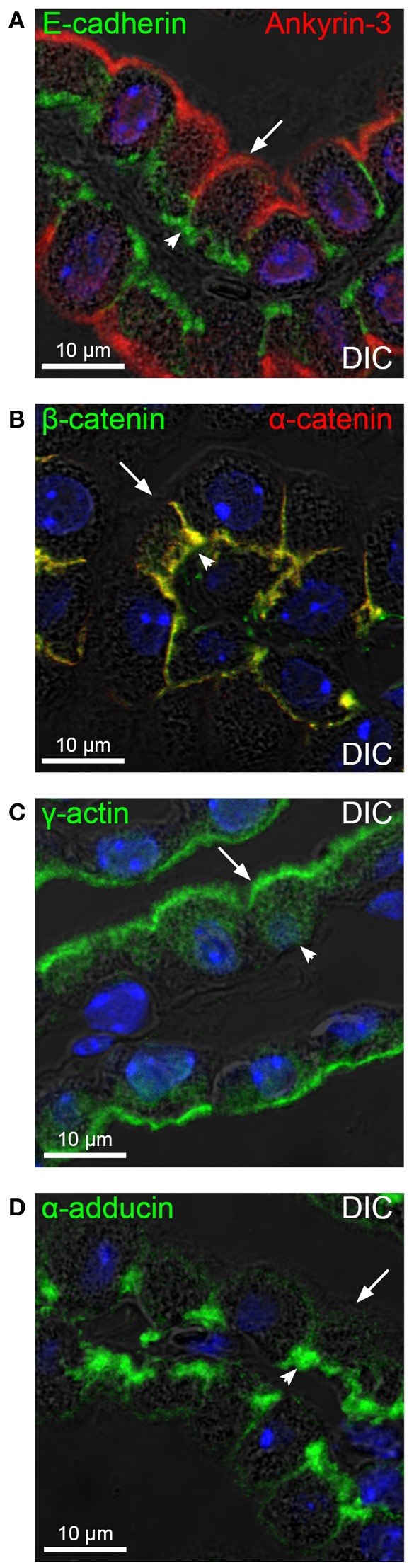
**Expression of E-cadherin, catenins, γ-actin, and α-adducin in the CPE**. Mouse brain sections were immunostained for E-cadherin, β-catenin, γ-actin, and α-adducin (in green), as well as ankyrin-3 and α-catenin (in red). **(A)** Immunohistochemical double immunolabeling for E-cadherin and ankyrin-3 overlayed on the corresponding DIC image. **(B)** Double immunolabeling for α- and β-catenin, overlaid on the corresponding DIC image. **(C,D)** Immunolabeling and DIC images for γ-actin and α-adducin, respectively. Arrows indicate the luminal membrane, while arrowheads indicate the basolateral membrane. Cell nuclei were visualized by Topro nuclear staining (blue).

The basolateral adhesion molecule E-cadherin is known to link the cytoskeleton through either ankyrins or catenins. From Figure 1B (right panel), it appears that ankyrin-3 would be an unlikely anchor for E-cadherin as it is located near the luminal membrane. Figure 2A shows that E-cadherin localizes to the basolateral membrane domain of the CPE, opposite ankyrin-3 in the luminal membrane domain. As shown in Figure 2B, α- and β-catenin immunoreactivity is also pronounced in the basolateral domain. Both α-catenin and β-catenin is known to link to the actin cytoskeleton. Figure 2C shows a predominant luminal membrane domain immunolabeling for γ-actin, which extends into the microvilli. Thus, in the CPE, it seems more likely that E-cadherin is linked to the general actin cytoskeleton through catenins rather than through ankyrin-3 and spectrins.

### The choroid plexus displays a typical epithelial adducin expression pattern

**Figure 3:**
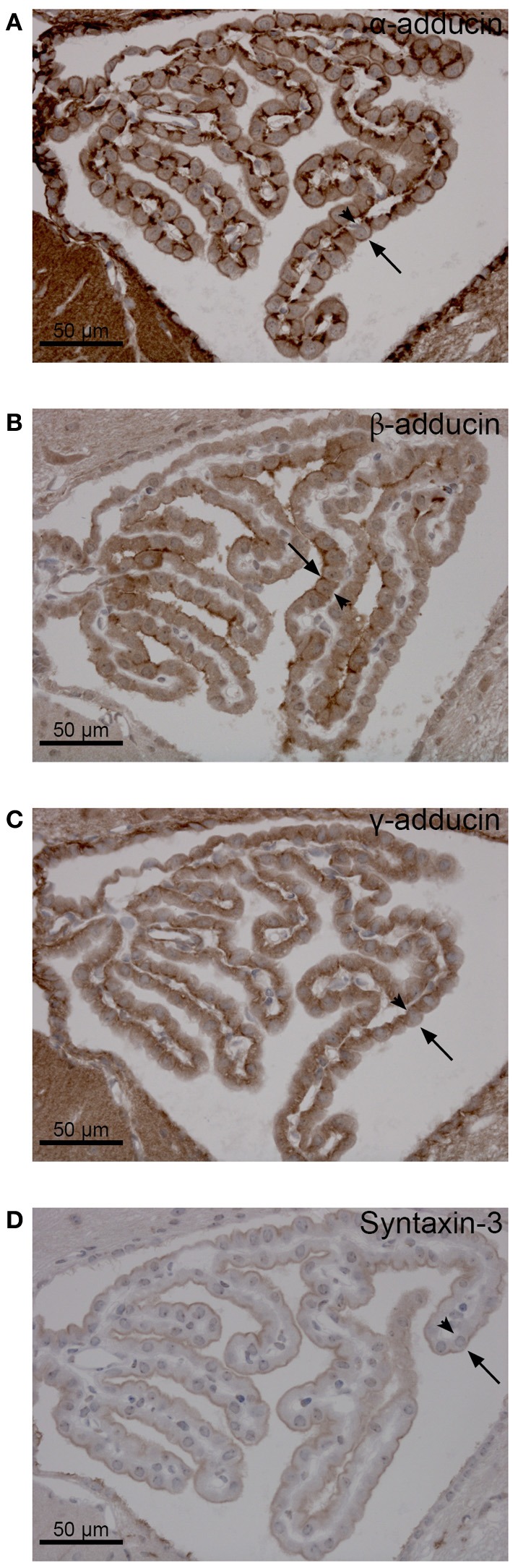
**Distribution of α-, β-, and γ-adducin in the mouse CPE. (A–C)** Peroxidase immunohistochemical localization of α-, β-, and γ-adducin in the normal mouse CPE, as indicated. **(D)** Immunostaining of the normal mouse choroid plexus for syntaxin-3. Arrows indicate the luminal membrane, while arrowheads indicate the basolateral membrane.

Adducins are alternatives to ankyrins for linking AE2 to the cytoskeleton at the basolateral membrane of the choroid plexus, as they co-sediment separately from ankyrin and the Na^+^,K^+^-ATPase in sucrose gradients (Marrs et al., 1993). Adducins bind both the spectrin and actin cytoskeleton and adducin immunoreactivity was previously shown in proximity to AE2 in the CPE (Alper et al., 1994). Adducin forms dimers consisting of α-β or α-γ subunits. The brain sections were immunostained for the three adducin forms in order to establish their relative locations in the CPE. The α-adducin immunoreactivity was observed near both the luminal and basolateral membrane (Figures 2D, 3A), with highest reactivity at the basal labyrinth as expected.

Immunofluorescence staining for β-adducin and γ-adducin failed, but peroxidase stained sections are seen in Figures 3B,C, respectively. Antibodies against β-adducin yielded staining in the luminal domain, while γ-adducin staining was more pronounced toward the basolateral cell domain. Despite the variable performance of the adducin antibodies, the cellular distribution of adducins in CPE seems comparable to what is observed in renal epithelia (not shown). Syntaxin-3 is a typical luminal membrane SNARE protein (Mellman and Nelson, 2008), which ascertains the insertion of specific vesicles destined to this part of the plasma membrane. Figure 3D shows that syntaxin-3 is also a luminal membrane protein in CPE. Commercially available antibodies against the basolateral SNARE protein syntaxin-4 did not produce reliable immunostaining in CPE or control epithelia (not shown).

### Moesin does not redistribute to the basolateral membrane in the choroid plexus of *slc4a10* ko mice

**Figure 4:**
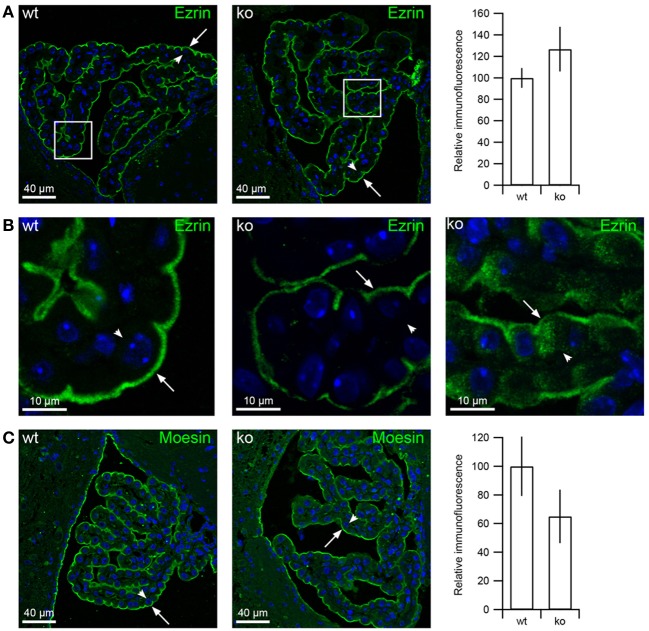
**Ezrin and moesin expression in CPE from *slc4a10* wt and ko mice**. Mouse brain sections were immunofluorescence stained with ezrin and moesin antibodies (in green). **(A)** Immunohistochemical detection of ezrin in *slc4a10* wt and ko, as indicated. Bar graph on the right show the semi-quantitation of the ezrin immunofluorescence in *slc4a10* wt and ko mouse CPE (wt: *n* = 5, ko: *n* = 4). **(B)** Immunohistochemical detection of ezrin at a higher magnification in *slc4a10* wt and ko, as indicated. **(C)** Immunostaining for moesin in *slc4a10* wt and ko, respectively. Bar graph on the right show the semi-quantitation of the moesin immunofluorescence in *slc4a10* wt and ko mouse CPE (wt: *n* = 5, ko: *n* = 4). Arrows indicate the luminal membrane, while arrowheads indicate the basolateral membrane. Cell nuclei were visualized by Topro nuclear staining (blue).

**Figure 5:**
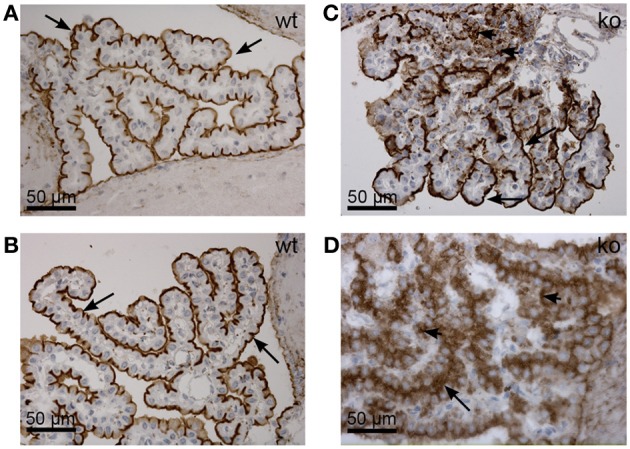
**Distribution of ezrin in the choroid plexus epithelium of *slc4a10* wt and ko mice. (A–D)** Labeling of choroid plexus brain sections with peroxidase conjugated anti-ezrin antibodies from *slc4a10* wt **(A,B)** and ko mice **(C,D)**. The two examples from *slc4a10* ko mice indicate the range of ezrin expression. Arrows indicate the luminal membrane, while arrowheads indicate the intracellular distribution of ezrin in *slc4a10* ko.

Previously, we have characterized the *slc4a10* ko mouse (Jacobs et al., 2008) and showed that the cellular localization of NHE1 in these mice was found in the basolateral plasma membrane instead of in the luminal membrane as in the *slc4a10* wt mouse (Damkier et al., 2009). Therefore, antibodies reacting specifically against the ezrin and moesin proteins known to interact with NHE1 directly or indirectly were used for immunohistochemical analysis of CPE. As previously observed, ezrin staining is found in the cytoplasm close to the luminal plasma membrane in the wt CPE (Figure 4A, micrographs). Ezrin staining was more variable among *slc4a10* ko mice, but was also here found predominantly in the subluminal domain in the CPE as assessed by immunofluorescence (Figure 4B). Figure 5 shows that the ezrin distribution appears more cytosolic in *slc4a10* ko mice compared to *slc4a10* wt in peroxidase stained sections from the same mice. Some labeling is observed in the luminal membrane domain, but the labeling is also found in intracellular compartments. The bar graph in Figure 4A shows that the semi-quantified immunofluorescence signal for ezrin did not differ between *slc4a10* wt and ko mice, (*p* = 0.556, *n* = 5 and 4 for wt and ko, respectively).

As shown in the micrographs of Figure 4C, moesin is found at the same cellular localization as ezrin, close to the luminal membrane, with no apparent difference in subcellular localization between the *slc4a10* wt and ko. The bar graph shows that the moesin signal did not differ quantitatively between *slc4a10* wt and ko mice (*p* = 0.286, *n* = 5 and 4 for wt and ko, respectively). Thus, neither ezrin nor moesin colocalizes with NHE1 in *slc4a10* ko mice.

### Decreased expression of β1-Na^+^,K^+^-ATPase but not ankyrin-3 in the *slc4a10* ko

**Figure 6:**
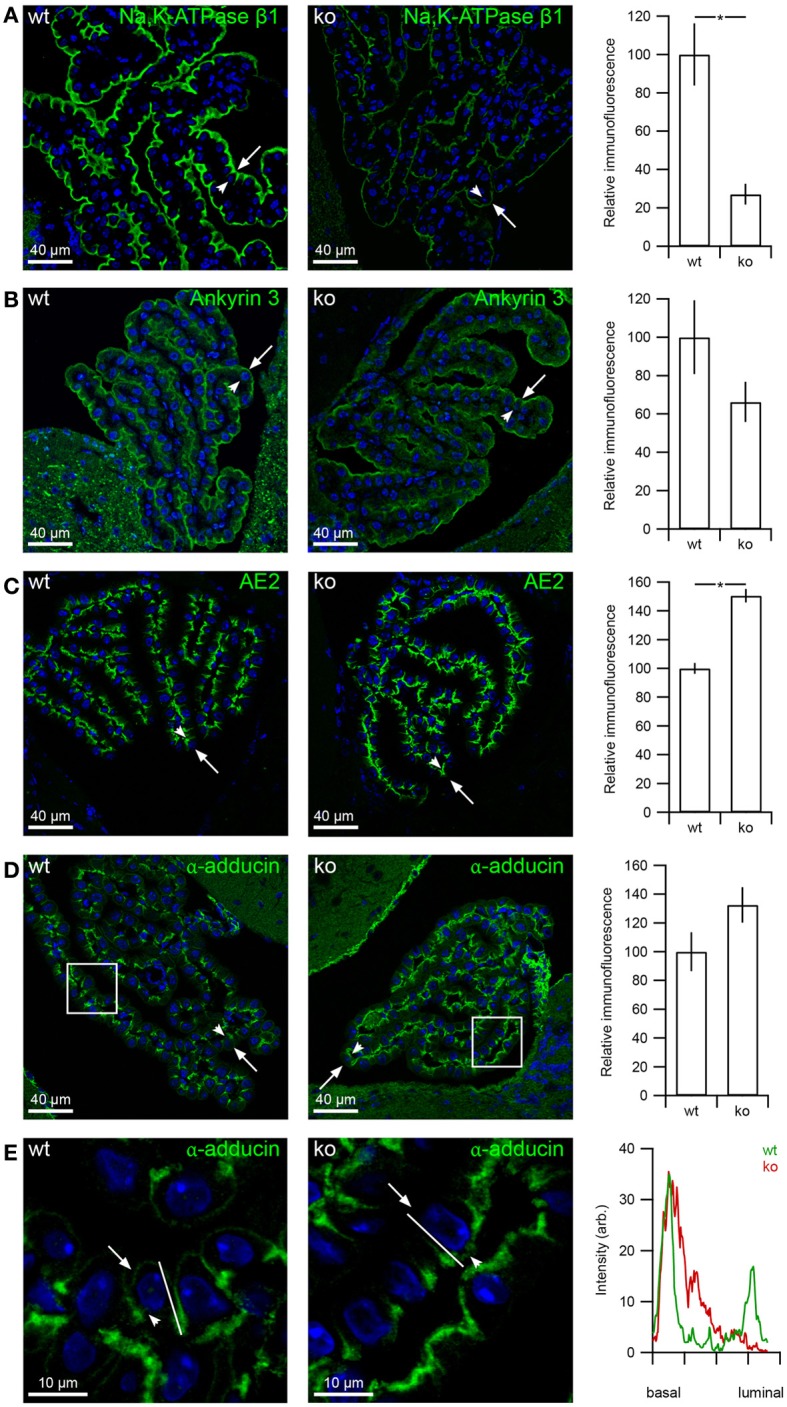
**β1 Na^+^,K^+^-ATPase and AE2 expression is changed in the CPE from *slc4a10* ko mice**. Mouse brain sections were immunostained for Na^+^,K^+^-ATPase β1 subunit, ankyrin-3, AE2, and α-adducin. **(A)** Immunohistochemical detection of β1-Na^+^,K^+^-ATPase in wt and ko, as indicated. Bar graphs on the right show the semi-quantitation of the respective immunofluorescence in *slc4a10* wt and ko mouse CPE (wt: *n* = 5, ko: *n* = 3). **(B)** Immunostaining for ankyrin-3 in *slc4a10* wt and ko, respectively. Bar graphs on the right show the semi-quantitation of the immunofluorescence in *slc4a10* wt and ko mouse CPE (wt: *n* = 5, ko: *n* = 4). **(C)** Immunohistochemical detection of AE2 in *slc4a10* wt and ko, as indicated. Bar graph on the right show the semi-quantification of the immunofluorescence in *slc4a10* wt and ko mouse CPE (*n* = 4). **(D)** Immunostaining for α-adducin in *slc4a10* wt and ko, respectively. Bar graphs on the right show the semi-quantitation of the immunofluorescence in *slc4a10* wt and ko mouse CPE (wt: *n* = 5, ko: *n* = 3). **(E)** Immunohistochemical detection of α-adducin in wt and *slc4a10* ko at a higher magnification. The histogram on the right is a linescan of the cellular fluorescence intensity from the basal to the luminal domain. The analyzed line is indicated on the two micrographs on the left. Arrows indicate the luminal membrane, while arrowheads indicate the basolateral membrane. ^*^indicates statistical significance. Cell nuclei were visualized by Topro nuclear staining (blue).

The Na^+^,K^+^-ATPase is linked to the spectrin cytoskeleton through ankyrin-3 in the choroid plexus (Marrs et al., 1993). The expression of α1-Na^+^,K^+^-ATPase was greatly decreased in *slc4a10* ko mice (Damkier and Praetorius, 2012). Therefore, it is feasible that the Na^+^,K^+^-ATPase β subunit as well as the anchoring proteins would display similar changes in abundance or in subcellular distribution. Figure 6A illustrates that the subcellular localization of the β1-Na^+^,K^+^-ATPase subunit is strictly luminal in CPE from both *slc4a10* wt and ko mice, and that the abundance is ~75% decreased in *slc4a10* ko CPE (Figure 6A bar graph, *p* = 0.0357, *n* = 5 and 3 for wt and ko, respectively). Ankyrin-3 abundance seemed lower in *slc4a10* ko CPE compared to wt littermates (Figure 6B, micrographs). This observation was not paralleled by a significant decrease in ankyrin-3 expression in *slc4a10* ko (Figure 6B bar graph, *p* = 0.250, *n* = 5 and 3 for wt and ko, respectively). Thus, the β1-Na^+^,K^+^-ATPase parallels the α1-Na^+^,K^+^-ATPase protein abundance and localization in the CPE of *slc4a10* wt and ko mice, while the protein abundance of their colocalizing scaffolding protein ankyrin-3 is unaffected in *slc4a10* ko CPE.

### AE2 abundance in the choroid plexus is increased in *slc4a10* ko mice

The expression and localization of Na^+^-transporting *slc4* gene family members NBCn1 and NBCe2 did not change significantly in *slc4a10* ko mice (Damkier et al., 2009). However, Figure 6C shows that the abundance of AE2 encoded by *slc4a2* is increased in the CPE from *slc4a10* ko mice relative to the wt mice. The semi-quantitation depicted in the bar graph indicates an ~50% increase in the relative AE2 abundance in *slc4a10* ko compared to wt (*p* = 0.029, *n* = 4 for wt and ko). The increase in AE2 protein was not accompanied by a significant change in α-adducin abundance in the *slc4a10* ko CPE (Figure 6D, *p* = 0.250, *n* = 5 and 3 for wt and ko, respectively). Nevertheless, we noted a non-significant trend toward increased total α-adducin abundance and a more pronounced basolateral staining relative to the luminal staining intensity. The luminal immunoreactivity was almost completely absent in the *slc4a10* ko CPE (Figure 6E).

### Decreased αI but not αII, βI, and βII abundance in the *slc4a10* ko CPE

**Figure 7:**
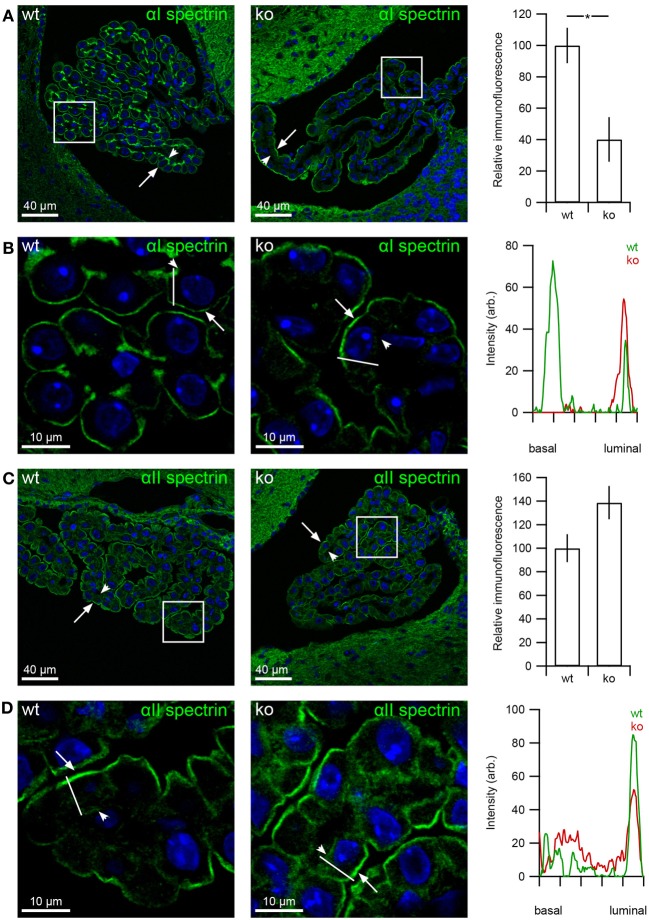
**Altered α1-spectrin expression in CPE from *slc4a10* ko mice**. Mouse brain sections were immunostained for αI- and αII-spectrin. **(A)** Immunolabeling for αI-spectrin in *slc4a10* wt and ko, as indicated. Bar graph on the right show the semi-quantitation of the respective immunofluorescence in *slc4a10* wt and ko mouse CPE (wt: *n* = 5, ko: *n* = 4). **(B)** High magnification micrograph of the subcellular αI-spectrin staining pattern. The graph is a linescan of the cellular fluorescence signal from the basal to the luminal domain. The analyzed line is indicated on the micrographs. **(C)** Immunolabeling for αII-spectrin in *slc4a10* wt and ko, as indicated. Bar graph on the right show the semi-quantitation of the respective immunofluorescence in *slc4a10* wt and ko mouse CPE (wt: *n* = 5, ko: *n* = 4). **(D)** High magnification micrograph of the subcellular αII-spectrin staining pattern. The graph is a linescan of the cellular fluorescence signal from the basal to the luminal domain. The analyzed line is indicated on the micrographs. Arrows indicate the luminal membrane, while arrowheads indicate the basolateral membrane. ^*^indicates statistical significance. Cell nuclei were visualized by Topro nuclear staining (blue).

The αI-spectrin immunoreactivity shows an ~60% decrease in protein abundance in the *slc4a10* ko compared to the wt (Figure 7A, *p* = 0.0357, *n* = 5 and 3 for wt and ko, respectively). This significant change in protein abundance was accompanied by a difference in cellular localization between the genotypes; from predominantly staining the basolateral labyrinth in the *slc4a10* wt to almost exclusively luminal expression in the *slc4a10* ko (Figure 7B). The immunofluorescence signal for αII-spectrin was not significantly altered in the CPE from *slc4a10* ko mice as compared to wt (Figure 7C, *p* = 0.143, *n* = 5 and 3 for wt and ko, respectively). There was no apparent change in cellular localization of the protein apart from more cytosolic immunoreactivity (Figure 7D).

**Figure 8:**
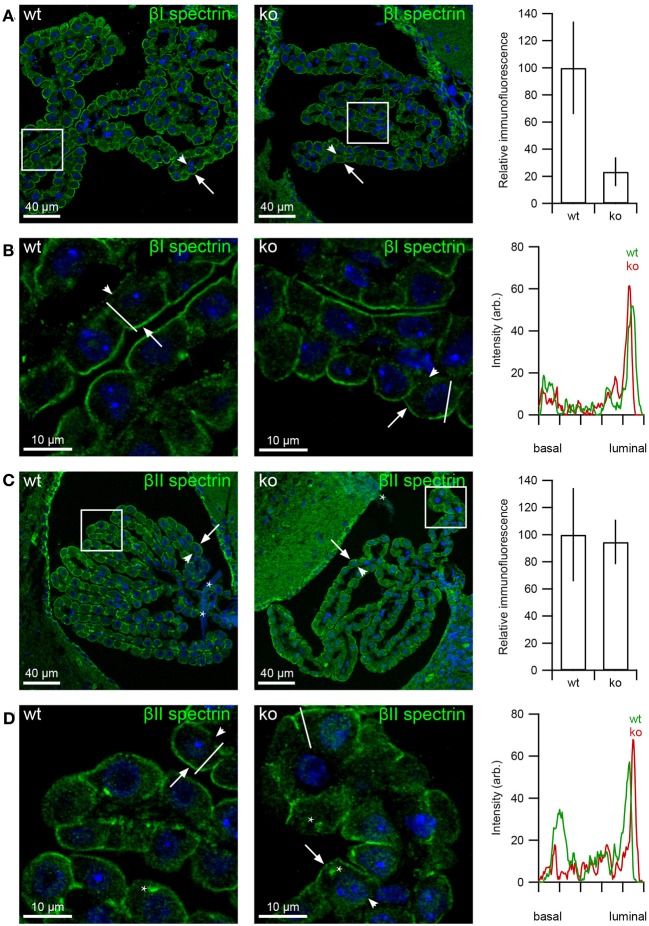
**Altered β-spectrin expression in CPE from *slc4a10* ko mice**. Mouse brain sections were immunostained for βI- and βII-spectrin. **(A)** Immunolabeling for βI-spectrin in *slc4a10* wt and ko CPE, as indicated. Bar graph on the right show the semi-quantitation of the respective immunofluorescence in *slc4a10* wt and ko mouse CPE (wt: *n* = 5, ko: *n* = 4). **(B)** High magnification micrograph of the subcellular βI-spectrin staining pattern. The graph is a linescan of the cellular fluorescence signal from the basal to the luminal domain. The analyzed line is indicated on the micrographs. **(C)** Immunolabeling for βII-spectrin in *slc4a10* wt and ko, as indicated. Bar graph on the right show the semi-quantitation of the respective immunofluorescence in *slc4a10* wt vs. ko mouse CPE (wt: *n* = 5, ko: *n* = 4). ^*^ denotes artifacts. **(D)** High magnification micrograph of the subcellular βII-spectrin staining pattern. ^*^ denotes supra-nuclear labeling. The graph is a linescan of the cellular fluorescence signal from the basal to the luminal domain. The analyzed line is indicated on the micrographs. Arrows indicate the luminal membrane, while arrowheads indicate the basolateral membrane. Cell nuclei were visualized by Topro nuclear staining (blue).

Analysis of the βI-spectrin abundance in the CPE resulted in a non-significant decrease in the *slc4a10* ko compared to wt mice (Figure 8A, *p* = 0.057, *n* = 5 and 3 for wt and ko, respectively), where the cellular localization did not change markedly (Figure 8B). The immunofluorescence signal for βII-spectrin shows no difference between *slc4a10* ko and wt CPE (Figure 8C, *p* = 0.85, *n* = 5 and 3 for wt and ko, respectively), whereas the cellular localization appears less basolateral in the ko mice (Figure 8D).

### E-cadherin and β-catenin abundance does not change in the choroid plexus of *slc4a10* ko mice

**Figure 9:**
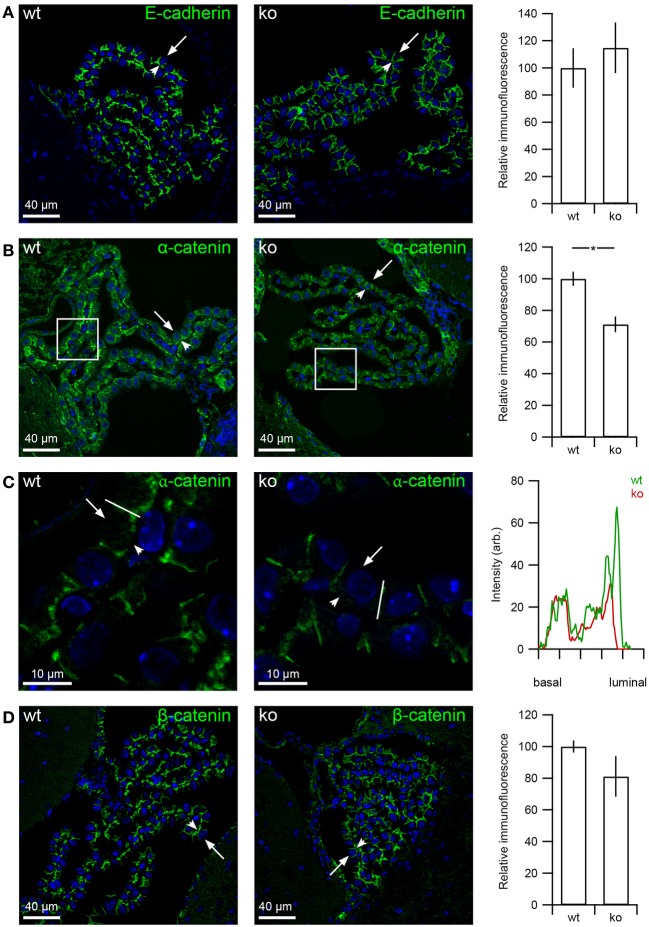
**E-cadherin, α- and β-catenin expression in CPE from *slc4a10* wt and ko mice**. Mouse brain sections were immunostained for E-cadherin and α- and β-catenin, as indicated. **(A)** Immunohistochemical detection of E-cadherin in *slc4a10* wt and ko, as indicated. Bar graphs on the right show the semi-quantitation of the immunofluorescence in *slc4a10* wt and ko mouse CPE (*n* = 4). **(B)** Immunolabeling for α-catenin in *slc4a10* wt and ko, as indicated. Bar graph on the right show the semi-quantitation of the immunofluorescence in *slc4a10* wt and ko mouse CPE (wt: *n* = 5, ko: *n* = 3). **(C)** High magnification micrograph of the subcellular α-catenin staining pattern. The histogram on the right is a linescan of the cellular fluorescence intensity from the basal to the luminal domain. The analyzed line is indicated on the two micrographs on the left. **(D)** Immunolabeling for β-catenin in *slc4a10* wt and ko, as indicated. Bar graph on the right shows the semi-quantitation of the immunofluorescence in wt and ko mouse CPE (wt: *n* = 5, ko: *n* = 3). Arrows indicate the luminal membrane, while arrowheads indicate the basolateral membrane. ^*^indicates statistical significance. Cell nuclei were visualized by Topro nuclear staining (blue).

Major changes in protein abundances of E-cadherin and the catenins were not expected in the *slc4a10* ko CPE, as the major changes in protein expression are observed in relation to the luminal plasma membrane. The micrographs in Figure 9A shows similar labeling intensity and cellular distribution of E-cadherin in *slc4a10* wt and ko CPE (*p* = 0.686, *n* = 4 and 4 for wt and ko). Nevertheless, as seen in Figure 9B, the abundance of α-catenin is significantly decreased in the *slc4a10* ko CPE as compared to the wt (Figure 9B Bar graph, *p* = 0.0357, *n* = 5 and 3 for wt and ko, respectively). Furthermore, the cellular localization of α-catenin seems to become more abundant in the lateral membrane domain and less in the basolateral labyrinth (Figure 9C). For β-catenin, similar expression patterns and protein abundances were observed in the two genotypes (Figure 9D, *p* = 0.143, *n* = 5 and 3 for wt and ko, respectively).

### Claudin-2 abundance in the choroid plexus epithelium is decreased in *slc4a10* ko mice

**Figure 10:**
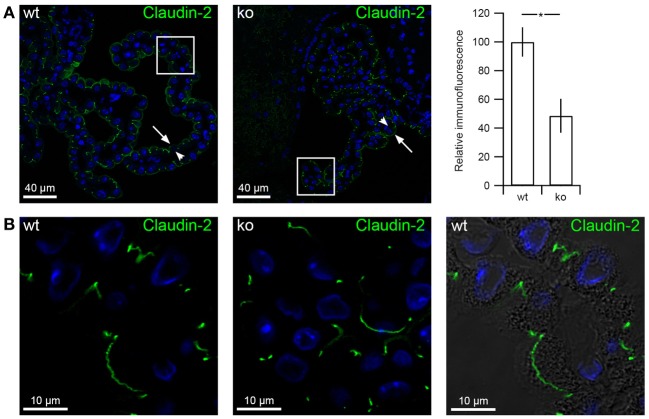
**Claudin-2 expression decreases in CPE from *slc4a10* ko mice. (A)** Immunohistochemical detection of claudin-2 in *slc4a10* wt and ko CPE, as indicated. Bar graph on the *right* show the semi-quantitation of the immunofluorescence in *slc4a10* wt and ko mouse CPE (wt: *n* = 5, ko: *n* = 3). **(B)** High magnification micrographs of claudin-2 immunoreactivity in *slc4a10* wt and ko CPE, as indicated. Arrows indicate the luminal membrane, while arrowheads indicate the basolateral membrane. ^*^indicates statistical significance. Cell nuclei were visualized by Topro nuclear staining (blue).

Claudin-2 expression in the tight junction of the CPE is most likely a determining factor in paracellular permeability in the epithelium. We hypothesized that decreased expression of ion and water transporters in CPE of *slc4a10* ko mice and most likely also decreased CSF secretion capacity would minimize the need for paracellular movement of ions and water molecules through claudin-2. The representative micrographs in Figures 10A,B illustrate that the claudin-2 immunoreactivity in CPE is decreased in *slc4a10* ko compared to *slc4a10* wt mice. The occasional observations of continuous lines of claudin-2 labeling in the luminal cell domain probably corresponds to tangentially sectioned cells, as judged from the DIC overlay in Figure 10B. The bar graph shows an ~50% decrease in CPE anti-claudin-2 staining in the *slc4a10* ko (*p* = 0.0357, *n* = 5 and 3 for wt and ko, respectively). Thus, it is likely that the capacity for selective movement of certain ions and perhaps water molecules via the paracellular route may be limited in *slc4a10* deficient mice.

## Discussion

**Figure 11:**
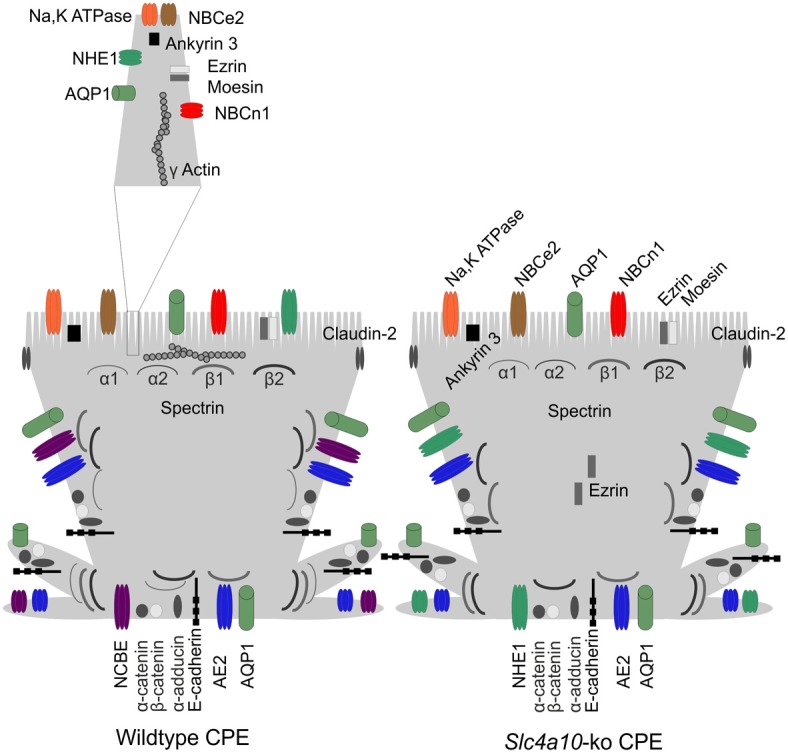
**Model of the subcellular position of membrane transporters, cytoskeletal components, and anchoring proteins in the CPE cell from normal and *slc4a10* knockout mice, as indicated**. The microvilli are indicated on the luminal membrane and the basal infoldings protrude laterally from the cell basis, separating the basal and the lateral membrane domains. See text for details.

Previously, we have shown that disruption of the gene encoding the key Na^+^ loader, Ncbe, in the CPE leads to significant changes in both the organization and abundance of other transport proteins (Damkier et al., 2009; Damkier and Praetorius, 2012). Protein abundance of the Na^+^,K^+^-ATPase α1 subunit and AQP1 was markedly reduced in *slc4a10* ko, and NHE1 was localized to the basolateral membrane as opposed to the luminal membrane. However, the other Na^+^HCO^−^_3_ cotransporters NBCn1 and NBCe2 were unaffected in the *slc4a10* ko mice. In the current study, we further explored the effects in the *slc4a10* ko mice; on the cellular localization and abundance of proteins expected to be involved in the polarization of CPE cells, with emphasis on anchoring and cytoskeletal proteins. Table 2 and Figure 11 are summarizing the subcellular localization of the membrane-, anchoring-, and cytoskeletal proteins located in this and previous studies. We report that AE2 protein abundance is significantly increased in the *slc4a10* ko mice and that αI-spectrin protein, which is present mainly in the basolateral membrane in the wt, is almost exclusively located in the apical membrane in the ko. Additionally, the protein abundance of αI-spectrin is decreased in the *slc4a10* ko CPE. Furthermore, we show that the protein levels of both claudin-2 and α-catenin are decreased in *slc4a10* ko as compared to wt.

**Table 2:** **Summary of data presented for CPE in the current and two previous studies (Damkier et al., 2009; Damkier and Praetorius, 2012)**.

	***Slc4a10***	**Basal**	**Lateral**	**Labyrinth**	**Luminal**
**MEMBRANE PROTEINS**					
α1 Na,K-ATPase	wt				+++
	ko				+
β1 Na,K-ATPase	wt				+++
	ko				+
AE2	wt	+	+	+	
	ko	++	++	++	
NCBE	wt	+	+	+	
	ko				
NHE1	wt				+
	ko	+	+	+	
NBCn1	wt				+
	ko				+
NBCe2	wt				+
	ko				+
AQP1	wt	++	++	++	+++
	ko	+	+	+	+
**ANCHORING PROTEINS**					
Ankyrin-3	wt				+
	ko				+
α-catenin	wt		+	++	
	ko		+	+	
β-catenin	wt		+	+	
	ko		+	+	
α-adducin	wt	+	+	++	+
	ko	+	+	+++	
Ezrin	wt				+
	ko				+
Moesin	wt				+
	ko				+
**CYTOSKELETAL PROTEINS**					
α1-spectrin	wt	+	+	++	+
	ko				++
α2-spectrin	wt		+	+	++
	ko		+	+	++
β1-spectrin	wt		+	+	++
	ko		+	+	+
β2-spectrin	wt	+	+	+	++
	ko		+	+	+
**CELL-CELL INTERACTIONS**					
Claudin-2	wt				++
	ko				+
E-cadherin	wt	+	+	++	
	ko	+	++	+	

The subcellular sites basal, lateral, labyrinth, and luminal cover both the respective parts of the plasma membrane proteins (transporters and cell-cell interaction proteins) and the sub-membrane part of the cytoplasm for soluble cytosolic proteins (anchoring and cytoskeletal proteins).

Marrs and Alper and their respective co-workers reported that the expression of both ankyrin and spectrin in the CPE was most prominent in the luminal membrane domain of the cells (Marrs et al., 1993; Alper et al., 1994). Here, we show that the mouse CPE expresses only ankyrin-3 and the αI-, αII-, βI-, and βII-spectrins. The identification of αII- and βII-spectrins was anticipated, whereas αI- and βI-spectrins are considered erythroid spectrin forms with additional expression of certain variants in the brain (Machnicka et al., 2012). Ankyrin-3 immunoreactivity was observed in accordance with the two previous studies, and alongside three of the spectrins. However, αI-spectrin was expressed both in the luminal and in the basolateral membrane domain, especially near the basal labyrinth. As spectrins form heterodimers of α and β subunits, our data suggest that both βI and βII could dimerize with αI-spectrin at its basolateral localization although the immunoreactivity for both β subunits is weak at this site. We note that ankyrin-3 immunoreactivity, as expected, protrudes into the microvilli of the CPE, where it anchors the Na^+^,K^+^-ATPase and other membrane proteins to the spectrin cytoskeleton. By contrast, none of the spectrin forms were localized to the microvilli and seemed restricted to a subluminal position most likely corresponding to the terminal web. Thus, we are intrigued by the lack of morphological evidence for supposed interaction of ankyrin-3 with spectrin in CPE. In this particular epithelium, the anchoring of luminal membrane proteins to the spectrin cytoskeleton via ankyrin-3 (and other anchoring proteins) appears to be indirect. One might speculate that ankyrin-3 in CPE binds spectrin via microvillar filamentous actin, as γ-actin staining stretches from the terminal web into these structures.

In order to explain the spatial separation of a basolateral cadherin and an unusually located spectrin in the luminal domain, Marrs and colleagues suggested that the choroid plexus expressed B-cadherin instead of the epithelial isoform, E-cadherin (Marrs et al., 1993). We here confirm recent reports on the expected expression and basolateral localization of E-cadherin in CPE (Lobas et al., 2012). Nonetheless, E-cadherin is now believed mainly to link to the actin cytoskeleton via the catenins and actinin, rather than the mainly luminal domain spectrins (Nelson, 2008). Our results are in accordance with such an organization, where E-cadherin is unaffected by the disappearance of the basolaterally expressed αI-spectrin. Here, γ-actin was predominantly expressed in the luminal domain, but previous studies have found ample expression of non-myocyte and f-actin forms more broadly in the CPE cells forming a base for immobilization of basolateral membrane proteins (Marrs et al., 1993; Alper et al., 1994; Li et al., 2009). Also, it seems reasonable to state that the adducins take their usual epithelial positions (Baines, 2010) in the CPE, with luminal domain α/β dimers and basolateral α/γ dimers. We note that E-cadherin is not confined to adherens junctions as described widely (Alberts et al., 2007); it clearly appears to be involved more broadly in cell-cell and cell-matrix interaction. This issue may need further attention from future investigations.

We previously reported a partial disruption of the luminal domain expression of ezrin in CPE from *slc4a10* ko mice (Damkier et al., 2009). Here, we extend the finding and show that another protein of the ERM complex, moesin, does not redistribute in the same cells. Moesin and ezrin bind membrane proteins or their anchor/scaffolding proteins as well as actin. The explanation for the different staining pattern of these ERM proteins may lie in either of the following characteristics: (1) As opposed to ezrin, moesin has been reported to bind microtubules in addition to actin (Solinet et al., 2013), and (2) only ezrin binds the sodium-hydrogen exchanger regulatory factor (NHERF) (Cha and Donowitz, 2008). The latter is a plausible cause for the more cytosolic ezrin distribution, as the basolateral NHE1 expression in *slc4a10* ko CPE minimizes the need for ezrin near the luminal membrane.

The abundance of β1-Na^+^,K^+^-ATPase in *slc4a10* ko CPE was decreased to the same extent as we previously reported for the α1 subunit (Damkier and Praetorius, 2012). Ankyrin-3 is most likely a link between the Na^+^,K^+^-ATPase complex and the cytoskeleton in CPE. However, the abundance of ankyrin-3 was not affected by a parallel decrease and remained in the brush border of the epithelium. We speculate that ankyrin-3 is more abundant at the luminal plasma membrane than the Na^+^,K^+^-ATPase in *slc4a10* ko, as it plays a role in anchoring other membrane proteins to the actin/spectrin cytoskeleton, such as NKCC1 and NBCe2. The abundance of the Na^+^ independent Cl^−^/HCO^−^_3_ exchanger AE2 in *slc4a10* ko was not studied previously. We find a surprising higher abundance of AE2 in the *slc4a10* ko, although still with a basolateral localization. The higher AE2 protein level in *slc4a10* ko is accompanied by a non-significant trend toward increased α-adducin expression. Furthermore, the expression of α-adducin seems more pronounced in the basolateral domain, and less apparent in the luminal domain. These findings suggests that the adducin scaffold is altered in the *slc4a10* ko CPE compared to the wt, and perhaps consequently increases the stability of AE2 in the basolateral membrane.

The spectrins form a part of the cytoskeleton and also link membrane proteins or their anchor/scaffolding proteins to actin filaments (Baines, 2010). In *slc4a10* ko CPE, the distribution of αI-spectrin is profoundly changed from mainly in the basolateral domain to almost exclusively in the luminal domain. This change is observed in parallel to a significant reduction in αI-spectrin abundance. In contrast to αI-spectrin, the cellular localization of αII-spectrin seems more cytoplasmic or basolateral in *slc4a10* ko. Thus, it seems that the loss of one α-spectrin leads to a replacement with another α-spectrin in the basolateral domain, presumably to compensate partly for a decrease in capacity of binding membrane proteins to the spectrin cytoskeleton. The protein abundance of αII-, βI, and βII-spectrins in the CPE show no significant change when comparing *slc4a10* wt and ko. The mainly luminal domain βI-spectrin shows no apparent cellular re-distribution, while the βII-spectrin basolateral localization seems slightly decreased.

We speculate that in the basolateral domain it is predominantly βII-spectrin that dimerizes with αI-spectrin and that these spectrins are not required for retaining the proposed adducin-bound AE2 in the basolateral membrane. With regards to the decrease of luminal Na^+^,K^+^-ATPase and AQP1 in *slc4a10* ko CPE, it is unlikely that changes in spectrins are a primary event in this dysregulation; both α-spectrins are maintained in the luminal domain in *slc4a10* ko CPE, and only βI-spectrin is decreased to a similar degree as the Na^+^,K^+^-ATPase subunits. Interestingly, comparable changes in the CPE expression of Na^+^,K^+^ATPase subunits, Ncbe (*slc4a10* gene product), and βII-spectrin have been reported in *slc4a5* ko mice (Kao et al., 2011). As the spectrins form heterodimers (Baines, 2010) it is tempting to suggest that a third type of β-spectrin is expressed in the luminal domain of *slc4a10* ko CPE. However, we observed no β-spectrins in the cells apart from βI and βII.

E-cadherin abundance in CPE was unaffected by *slc4a10* ko. However, the catenin binding partners seemed slightly less abundant in the ko mice with only α-catenin reaching statistical significance. Thus, these proteins do not appear to maintain a fixed stoichiometry in CPE, just as we noted for the hypothesized interactions between Na^+^,K^+^-ATPase/ankyrin-3, NHE1/ezrin/moesin, AE2/α-adducin, and α-spectrins/β-spectrins. These proteins are not distributed or regulated in parallel with their normal binding partners. In contrast to the stable maintenance of E-cadherin in the cell-cell and cell-matrix interactions, claudin-2 in the tight junctions was less abundant in the *slc4a10* ko CPE. As claudin-2 is involved in transepithelial Cl^−^ and H_2_O transport (Rosenthal et al., 2010), this suggests that the paracellular permeability of Cl^−^ and perhaps even H_2_O is reduced in the ko mice. Both properties would tend to decrease any direct or indirect contribution of paracellular transport to CSF secretion rate (Damkier et al., 2013).

The current investigation does not explain why *slc4a10* ko mice do not develop a normal expression level and cellular distribution of spectrins in the CPE. However, it is tempting to speculate that Ncbe recruits αI/βII spectrin to the basolateral membrane domain, especially to the basal labyrinth. In the *slc4a10* ko CPE, loss of basolateral domain accumulation of αI/βII spectrin may induce secondary changes in specific parts of the cytoskeleton and anchor proteins that mainly alter luminal membrane proteins AQP1 and Na^+^,K^+^-ATPase, while other proteins at that site, such as NBCn1 and NBCe2, are maintained. Testing this model experimentally seems conceivable using CPE cell culture systems, inducible *slc* gene knockout, or siRNA mouse models.

In conclusion, the current study does not explain the atypical polarization of the CPE, but we present findings that are inconsistent with fundamental cell biological paradigms developed in epithelial cell types other than CPE. The observations allow the generation of new hypotheses that can be tested experimentally in future studies. Because of the discrepancy between the regulation or cellular distribution among membrane proteins and their usual binding proteins in the CPE, further investigations should focus beyond known protein-protein interactions.

### Conflict of interest statement

The authors declare that the research was conducted in the absence of any commercial or financial relationships that could be construed as a potential conflict of interest.
